# Unintended Avulsion of Hypertrophic Adenoids in Posterior Nasopharynx: A Case Report of a Rare Complication Caused by Nasotracheal Intubation

**DOI:** 10.1155/2014/980930

**Published:** 2014-06-25

**Authors:** Hao-Hu Chen, Li-Chuan Chen, Yu-Hui Hsieh, Mao-Kai Chen, Chung-Ho Chen, Kuang-I Cheng

**Affiliations:** ^1^Department of Anesthesiology, Kaohsiung Medical University Hospital, Kaohsiung 807, Taiwan; ^2^Department of Oral and Maxillofacial Surgery, College of Dental Medicine, Kaohsiung Medical University, Kaohsiung 807, Taiwan; ^3^Faculty of Medicine, Department of Anesthesiology, College of Medicine, Kaohsiung Medical University, Kaohsiung 807, Taiwan

## Abstract

The enlarged adenoid serves as a mechanical obstacle on the nasopharynx to intricate nasotracheal intubation. No matter what video or direct laryngoscopic techniques are applied, nasotracheal tube navigation from the nasal valve area through the nasal cavity to the nasopharynx is always blind; trauma is not uncommon. Here we report a case of unintended avulsed adenoids that plugged the tube tip while the nasotracheal tube blindly navigated through the nasopharyngeal space. After failing to insert a bent tip of gum elastic bougie passing through the nasopharynx, an alternative method of NTI was performed by mounting the nasotracheal tube on a fiberoptic bronchoscope. The nasotracheal tube was successfully railroaded along the insertion tube of the fiberscope to the trachea.

## 1. Introduction

The adenoids (pharyngeal tonsils) are lymphoid tissue in the upper posterior aspect of the nasopharynx, designed to process infections in the nose and throat. Though the cause of adenoid hyperplasia is not really understood, many facts such as repeated infections [1], chronic inflammation [2], allergic rhinitis [2, 3], and heavy cigarette smoking [4] all trigger its hypertrophy. The enlarged adenoid serves as a mechanical obstacle on the nasopharynx to intricate nasotracheal intubation. Nasotracheal intubation (NTI) is needed for elective oromaxillofacial surgery. No matter what video or direct laryngoscopic techniques for NTI are used, nasotracheal tube insertion from the nasal valve through the nasal cavity to the nasopharynx is always blind; trauma is not uncommon. Herein, we report a case of unintended avulsed adenoids that plugged the tube tip while the nasotracheal tube was blindly navigated through the nasopharyngeal space. After failure to insert a bent tip of gum elastic bougie passing through the nasopharynx, an alternative method of NTI was performed by mounting the nasotracheal tube on a fiberoptic bronchoscope. The nasotracheal tube was successfully railroaded along the insertion tube of the fiberscope to the trachea.

## 2. Case Report

A 40-year-old man, with American Society of Anesthesiologists physical status II, height 167 cm, weight 63.5 kg, was scheduled for wide excision of oral tissue leukoplakia on the right buccal mucosa and tongue. He had a white patch over the right buccal area for 3 years and his right tongue ulcer had appeared for one year. His history included hypertension and diabetes mellitus with regular treatment, allergic rhinitis, and a heavy cigarette smoker (2 packs per day). His radiologic and laboratory test findings were not abnormal except for slightly elevated C-reaction protein.

**Figure 1:**
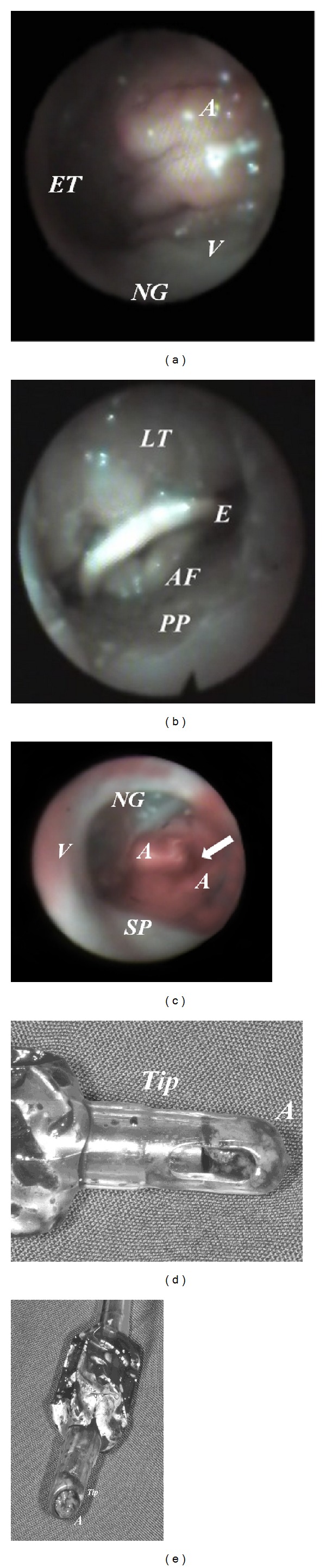
Hypertrophic adenoid avulsed by a nasotracheal tube. Hypertrophic adenoid viewing by a fiberscope (a). Hypertrophic lingual tonsil (b). Avulsed hypertrophic adenoid; a groove appear on the adenoid (c). Tip of the nasotracheal tube was filled with adenoid tissue but side hole was not occluded (d and e). A: adenoid hypertrophy; AF: arytenoid folds; E: epiglottis; ET: eustachian tube; LT: lingual tonsil; NG: nasogastric tube; PP: posterior pharyngeal wall; SP: soft palate; V: vomer.

An otolaryngologist examined his nasal cavities and selected his left nostril for nasotracheal intubation before he was taken to the operating room (OR). In OR, fentanyl (1 *μ*g/kg) and midazolam (0.03 mg/kg) were administered intravenously for sedation. An anesthesiologist examined his left nasal airway patency with a fiberscope. Turbinates were swollen, narrowing the nasal pathway, and nasopharynx with grade II adenoid hypertrophy [5] and oropharynx with lingual hyperplasia were found (Figures 1(a) and 1(b)). Four cotton-tipped applicators dipped with 6% cocaine were properly placed for at least five minutes for vasoconstriction and to blunt the branches of the trigeminal nerve under standard monitoring, including electrocardiogram, pulse oximetry, and noninvasive blood pressure measurement.

After that, intravenous induction agents including fentanyl 2 *μ*g/kg, thiopental 5 mg/kg, and propofol 1 mg/kg were intravenously administered. Mask ventilation was easily performed uneventfully. Nasotracheal intubation was facilitated with 0.6 mg/kg of rocuronium; the nasotracheal tube (RAE Nasal, Mallinckrodt Medical Athlone, Ireland of 7.0 inner diameter) was thermosoftened and lubricated with 2% lidocaine gel coating its tip and cuff.

The nasotracheal tube was inserted with bevel of the tube facing medially, and the tube passed through the nasal pathway along the space between the inferior turbinate and the floor of the nose. Mild resistance was encountered during tube insertion through the nasal pathway and on the nasopharyngeal space. However, a mass was filled with inlet of the tube as the tip of the nasotracheal tube was on the oropharynx, (Figure 1(d)). The plugged tube was withdrawn. Then, viewing from a fiberscope, a groove appeared on the hypertrophic adenoid (Figure 1(c)) and a bleeding point on the lower margin of the adenoid but not on the middle or inferior turbinate was found. After failure to insert a bent tip of gum elastic bougie passing through the nasopharynx from the selected nostril, we chose an alternative method of NTI by mounting the nasotracheal tube on a fiberoptic bronchoscope (outer diameter: 4 mm, working length: 600 mm, Olympus LF-2, Tokyo, Japan). The tip of the fiberscope was carefully passed through the nasal cavity and nasopharynx to the trachea. The nasotracheal tube was railroaded along the shaft of the fiberscope to the trachea. Surgical procedures were uneventful. The damaged retropharyngeal tissues did not bleed actively after operation as viewed by a fiberscope. Pathology of the specimen submitted tissue fragment of 0.5 × 0.2 × 0.1 showed eroded respiratory mucosa with exuberant lymphoid stroma. The patient recovered smoothly without obvious nasal pain, sore throat, or hoarseness.

## 3. Discussion

Nasal damage following NTI most frequently involves mucosa overlying the inferior turbinate and adjacent septum [6]. The most significant site of nasal obstruction is also at the nasal valve area or the anterior part of the inferior turbinate in the case of turbinate hypertrophy [7]. However, hypertrophic adenoid injuries during insertion of the nasotracheal tube through the nasopharyngeal space are an important but often neglected issue during conventional NTI. The hypertrophic adenoid is mainly composed of lymphoid tissues with a mound of cobblestone features. While the nasotracheal tube is blindly passing through the nasopharynx in a sharp curve, the posterior nasopharyngeal wall may impact its advancement. Hypertrophic adenoids are fragile and easily bleed during NTI.

In a case presentation, the unusual complication of avulsed adenoid might occur. The possible nasopharyngeal tissue damage during NTI includes retropharyngeal dissection [8], laceration [9], bleeding [8, 9], and tissues avulsion [10]. The nasotracheal tube advancing under a forward force avulsed hypertrophy adenoid not only induced tissue bleeding but also occluded the tube. Ng and Yew [10] reported that the avulsed adenoid that occluded the tube was not found till the critical point of nasotracheal tube into trachea, but ventilation could not occur. For early detection of hypertrophic adenoid not merely via fiberoptic bronchoscope or flexible nasopharyngoscopy, a noninvasive method with a lateral X-ray of the neck remains a reliable and valid diagnostic test [11].

Although avulsion of hypertrophic adenoid is an unusual complication, a routine assessment for patients with high risk is needed. In normal subjects without nasal obstruction, the prevalence incidence of hypertrophic adenoid is 55.1% and with nasal obstruction is 63.6% [12]. A history of allergic rhinitis [2] or being a heavy cigarette smoker [4] should alert the anesthetist to encounter potentially enlarged adenoids. Hypertrophic adenoids are often underestimated and ignored for those patients undergoing NTI. However, avulsion of hypertrophic adenoid is usually based on using improper pressure or forced advancement of the nasotracheal tube against opposing resistance on the nasopharynx. A groove on the hypertrophic adenoid, a bleeding point beneath it, and a lymphoid tissue filled with the tube tip can readily demonstrate the injured adenoid being cut by the nasotracheal tube.

In this case, after hypertrophic adenoid was avulsed, a blindly inserted gum elastic bougie failed because we were not able to navigate the soft bend with a fixed angle tip without resistance through the nasopharynx. The main reason is that the fixed tip of bougie did not match the curvature of the nasopharynx. Ng and Yew [10] also met the situation of adenoid occlusion from the nasotracheal tube, and orotracheal intubation was determined finally. However, a flexible fiberscope assisted by bleeding suction successfully went through the nasopharyngeal space in our case.

## 4. Conclusion

High-risk patients with allergic rhinitis and heavy smoking history should be assessed for abnormally enlarged lymphoid tissues to prevent unintentional avulsion hypertrophic adenoid during NTI. Hypertrophic adenoid injury should not be overlooked during NTI. However, under injured hypertrophic adenoid with bleeding, a flexible fiberoptic bronchoscope combined with a catheter bleeding suction is recommended for NTI.

## References

[1] Bernstein JM, Hasse E, Scannapieco F (2006). Bacterial interference of penicillin-sensitive and -resistant Streptococcus pneumoniae by Streptococcus oralis in an adenoid organ culture: implications for the treatment of recurrent upper respiratory tract infections in children and adults. *Annals of Otology, Rhinology and Laryngology*.

[2] Yildirim N, Şahan M, Karslioğlu Y (2008). Adenoid hypertrophy in adults: clinical and morphological characteristics. *Journal of International Medical Research*.

[3] Huang S-W, Giannoni C (2001). The risk of adenoid hypertrophy in children with allergic rhinitis. *Annals of Allergy, Asthma and Immunology*.

[4] Finkelstein Y, Malik Z, Kopolovic J, Bernheim J, Djaldetti M, Ophir D (1997). Characterization of smoking-induced nasopharyngeal lymphoid hyperplasia. *Laryngoscope*.

[5] Parikh SR, Coronel M, Lee JJ, Brown SM (2006). Validation of a new grading system for endoscopic examination of adenoid hypertrophy. *Otolaryngology—Head and Neck Surgery*.

[6] O’Connell JE, Stevenson DS, Stokes MA (1996). Pathological changes associated with short-term nasal intubation. *Anaesthesia*.

[7] Hilberg O (2002). Objective measurement of nasal airway dimensions using acoustic rhinometry: methodological and clinical aspects. *Allergy*.

[8] Krebs MJ, Sakai T (2008). Retropharyngeal dissection during nasotracheal intubation: a rare complication and its management. *Journal of Clinical Anesthesia*.

[9] Tintinalli JE, Claffey J (1981). Complications of nasotracheal intubation. *Annals of Emergency Medicine*.

[10] Ng SY, Yew WS (2006). Nasotracheal tube occlusion from adenoid trauma. *Anaesthesia and Intensive Care*.

[11] Lertsburapa K, Schroeder JW, Sullivan C (2010). Assessment of adenoid size: a comparison of lateral radiographic measurements, radiologist assessment, and nasal endoscopy. *International Journal of Pediatric Otorhinolaryngology*.

[12] Hamdan A-L, Sabra O, Hadi U (2008). Prevalence of adenoid hypertrophy in adults with nasal obstruction. *Otolaryngology—Head and Neck Surgery*.

